# Distractor Suppression When Attention Fails: Behavioral Evidence for a Flexible Selective Attention Mechanism

**DOI:** 10.1371/journal.pone.0126203

**Published:** 2015-04-27

**Authors:** James C. Elliott, Barry Giesbrecht

**Affiliations:** Department of Psychological and Brain Sciences, Institute for Collaborative Biotechnologies, University of California Santa Barbara, Santa Barbara, California, United States of America; University of Verona, ITALY

## Abstract

Despite consistent evidence showing that attention is a multifaceted mechanism that can operate at multiple levels of processing depending on the structure and demands of the task, investigations of the attentional blink phenomenon have consistently shown that the impairment in reporting the second of two targets typically occurs at a late, or post-perceptual, stage of processing. This suggests that the attentional blink phenomenon may represent the operation of a unique attentional mechanism that is not as flexible as other attentional mechanisms. To test whether the attentional blink is a fixed or flexible phenomenon, we manipulated first target task demands (i.e., difficulty) and measured the influence this had on processing a subsequently presented distractor and the second target. If the attentional blink represents a mechanism that is fixed and consistently fails at a single stage of processing, then manipulations of task difficulty should not affect distractor processing. However, if the attentional blink represents a more multifaceted and flexible mechanism, then task difficulty should modulate distractor processing. The results revealed that distractor processing during the AB was attenuated under high task difficulty. In addition, unlike previous studies, we failed to find a correlation between distractor processing and the severity of the attentional blink. Using a simulation, we demonstrate that the previously reported correlations may have been spurious and due to using variables that were not independent. Overall, the present results support the conclusion that the selectivity of attention during the AB is flexible and depends on the structure and demands of the task.

## Introduction

Given the complex dynamics of the external world, the human brain requires flexible mechanisms that can efficiently represent and process information required for successful goal-directed actions. Over a century’s worth of research has converged on the notion that selective attention is one such flexible and multifaceted mechanism that can operate at multiple levels of processing and in sensory, perceptual, and cognitive domains depending on the task at hand [1–6]. The idea that selective attention may influence processing at multiple levels was proposed at least as early as 1973 when Hillyard and colleagues [7] suggested that the extent to which attention modifies early auditory perceptual processing could be influenced by overall task difficulty. The finding that changing the structure or difficulty of the task can change the stage at which selective attention operates has since been verified by using both behavioral and neuroimaging methods in a variety of attention tasks and sensory modalities (for reviews see [2, 8]) suggesting the flexibility of the locus of selection is a fundamental property of attention [3, 5, 6, 9, 10].

In sharp contrast to the theoretical notion that selective attention is a flexible mechanism that can operate at multiple stages of processing, there is evidence that some attentional phenomena may not be so flexible. For instance, the attentional blink (AB) [11] is an impairment in identifying the second of two targets (T2) when it is presented within 200–500 ms after the first (T1) [11, 12]. Theories of the attentional blink (AB) phenomenon have consistently proposed that T2 processing is impaired after the semantic or categorical representation of the target has been extracted (for reviews see [13–15]). In other words, these models assume that selective attention, as measured during the AB, is rigid and operates only at a post-perceptual stage.

The assumption of a post-perceptual processing failure is a common theme in the corpus of AB theories and it was adopted because converging lines of behavioral and electrophysiological evidence indicated unimpaired categorical and semantic processing of both targets and distractors during the AB, despite the severe impairment in reportability [16–20]. The first evidence that target-related processing proceeded to post-perceptual stages during the AB was that the N400 event-related potential evoked by T2, which is an electrophysiological measure of semantic processing of the second target, was not modulated by the AB [16, 20]. Behavioral evidence comes from studies showing that personal names may not be subject to the AB [18] and even when T2 is identified incorrectly, it still can prime a subsequent related target (i.e., same identity, different case or semantically related) presented outside the AB [18].

Evidence that distractors are also processed to a post-perceptual stage was reported by Maki and colleagues [17], who used an RSVP of words and found that if the distractors presented between T1 and T2 were related to T2, performance was better than when the distractors were not related to T2. Interestingly, this effect was found to be independent of the AB, such that it was observed when the priming distractors and target were presented at short and long lags. Thus, this suggests that all information, including targets and distractors, are processed to a high level during the AB. More recently, the relationship between individual differences in distractor processing and the AB has also been used as a tool to investigate the post-perceptual processing failures that occur during the AB. Specifically, Dux and Marios [21] found that a priming distractor presented prior to T2 improved performance even when it was presented during the AB. It was also found that the magnitude of the AB was positively correlated with the influence of the prime, such that those individuals who exhibited a larger prime effect had a larger AB [21]. Furthermore, it was found that the prime effect was negatively correlated with T1 accuracy, suggesting that suppressing distractors improves T1 performance. These authors interpreted these results as evidence against theoretical accounts that suggest that distractor suppression is the cause of the AB (e.g. the Boost and Bounce Theory [15]), yet the results did not question the fundamental underlying assumption that all information is processed to a post-perceptual stage during the AB.

### The Current Study

The discrepancy between the notion that attention can act at multiple stages of information processing and theories of the AB could simply mean that the AB is a unique attentional phenomenon. While there are certainly unique aspects to the AB, there is no reason to assume that under appropriate task conditions the level at which processing is influenced by the AB would not be flexible. In fact, there is growing empirical evidence for flexible selection of targets during the AB [22–29]. While these studies are consistent with the notion that the AB represents a more flexible mechanism than current theories would suggest, the evidence comes from atypical AB tasks that often place a heavy emphasis on spatial selection and/or involve a switch in tasks between T1 and T2 [13]. Thus, the evidence for flexible selection that these studies provide may be due to the unique demands of the task rather than a fundamental property of the AB.

The main goal of the present work is to determine the influence of flexible selection on the processing of temporal distractors presented during the AB. In order to do so, we manipulated T1 task difficulty within the context of a standard two target RSVP task that was presented at fixation by simultaneously presenting 6 noise dots with T1 on half the trials. Importantly, this task did not place atypical demands on spatial selective attention compared to typical studies of the AB, nor did it require a task switch between T1 and T2. In this context, we assume that the flexibility of selective attention can be indexed by the extent to which information is processed to a post-perceptual level during the AB and, therefore, we measured the influence of a T2 distractor prime that was the same-identity (but different color) as T2 and that was present on half of the trials [21].

A secondary goal of the current study was to investigate the relationship between distractor processing and the magnitude of the AB. While Dux and Marios [21] reported a relationship between distractor processing and the AB, the manner in which this correlation was computed is potentially problematic. Specifically, to examine this relationship, Dux and Marios [21] calculated the prime effect as the difference between T2 percent correct at lag 4 when the prime was present and T2 percent correct at lag 4 when the prime was absent (lag 4 prime present—lag 4 prime absent) and calculated AB magnitude as the difference between T2 percent correct at lag 10 when the prime was absent and T2 percent correct at lag 4 when the prime was absent (lag 10 prime absent—lag 4 prime absent). While there is nothing inherently wrong with examining these different effects, it is problematic to make comparisons or examine relationships between these effects because both the AB magnitude and the prime effect calculations include the lag 4 prime absent condition, which mathematically inflates the shared variance between the two variables of interest (see S1 Appendix for a mathematical discussion of this issue). Other recent work has also adopted the same strategy of correlating the prime effect with AB magnitude at lag 4 [30]. Therefore, the current study explored whether or not the relationship between the prime effect and the AB magnitude would be observed when these effects are calculated independently. This was done both by conducting a simulation to determine if similar effects could be observed with random numbers and by calculating the prime effect and AB magnitude from independent subsets of data.

The above mathematical shortcoming, however, doesn’t question the finding that the presentation of a priming distractor improved performance on the T2 task, and this has been cited as evidence for post-perceptual processing of distractors during the AB. Thus, the key theoretical question of the current experiment is whether or not T1-difficulty decreases the extent to which the prime influences T2 accuracy. Finding that T1-difficulty decreases the influence of the prime would be consistent with the view that processing of information during the AB is flexible.

## Methods

### Participants

61 undergraduates from the University of California, Santa Barbara, participated in this experiment for course credit. Participants provided written informed consent and all procedures were approved by the Human Subjects Committee at the University of California, Santa Barbara. We applied a recursive outlier procedure [31] that excluded 10 participants with condition means that were more than 2.5 standard deviations away from the condition mean of the group. In order to ensure that the reported results were not merely due to the exclusion criterion, the analysis was also conducted excluding only one subject that had empty cell means due to poor T1 performance. The results of this analysis were consistent with what is reported.

### Apparatus and Stimuli

Targets and distractors were upper case letters presented on a gray screen in Arial size 32 font (.51° x .41°). T1 was red, T2 was green, and the distractors were white. The T1-noise dots that were presented on half the trials were randomly positioned within a .51° x .51° frame centered on T1 and were .07° in diameter. Stimuli were presented on a 19-inch color CRT monitor positioned 110 cm from the participant. Stimulus presentation, timing, and response acquisition were controlled using the Psychophysics toolbox [32].

### Procedure

**Fig 1:**
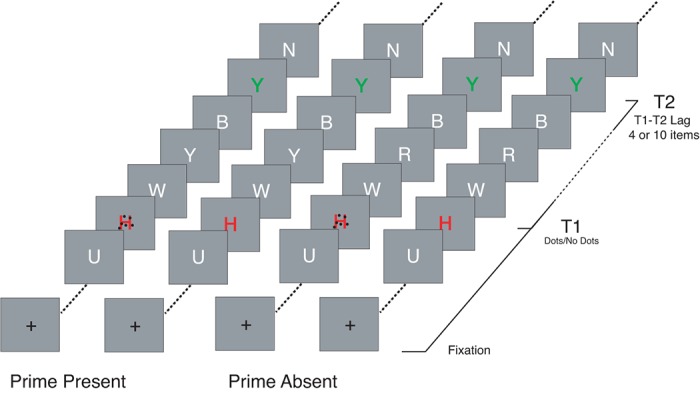
Schematic representation of the trial sequence. T1 was always red while T2 was green.

Participants initiated each trial by pressing the space bar. Trials began with a 500–1000 ms blank interval during which a fixation cross was on the screen, followed by the RSVP sequence (Fig 1). All items in the RSVP sequence were presented for 96 ms with no interstimulus interval. T1 was always the 10^th^ item. T2 was either the 4^th^ item after T1 or the 10^th^ item after T1 (i.e., lag 4 or 10). The prime, if present, was the second item after T1. After the final RSVP distractor, there was a 500–1000 ms blank interval, followed by the T1 and T2 response screens. Participants were instructed to identify each target by typing the identity using the keyboard. After the participant responded, a fixation cross appeared on the screen indicating that the next trial could be initiated when the participant was ready.

### Design

The design included three within subjects factors: T1-T2 lag (4 or 10), T1 task difficulty (dots or no dots), and T2 prime (present or absent). The task difficulty manipulation consisted of presenting 6 task irrelevant dots on top of the T1. The prime manipulation was used to assess the level of processing during the AB period (200–500 ms). When the prime was present, it was presented at lag 2 and, except for its color, the prime was identical to the T2 stimulus. All variables were factorially combined and randomly intermixed within a session. There were a total of 400 trials, presented in 10 blocks of 40 trials each.

## Results

The results are presented in three sections. In the first section, the influence of T1-difficulty on the processing of the distractor prime is reported. In the second section, we present the results of the correlation between the prime effect and the AB magnitude using methods that ensure the variables do not share variance. In the third section, a computational simulation exploring the influence of the calculations on the relationship between the prime effect and the AB magnitude is discussed.

### Influence of T1-difficulty on Distractor Processing

#### T1 Data

**Fig 2:**
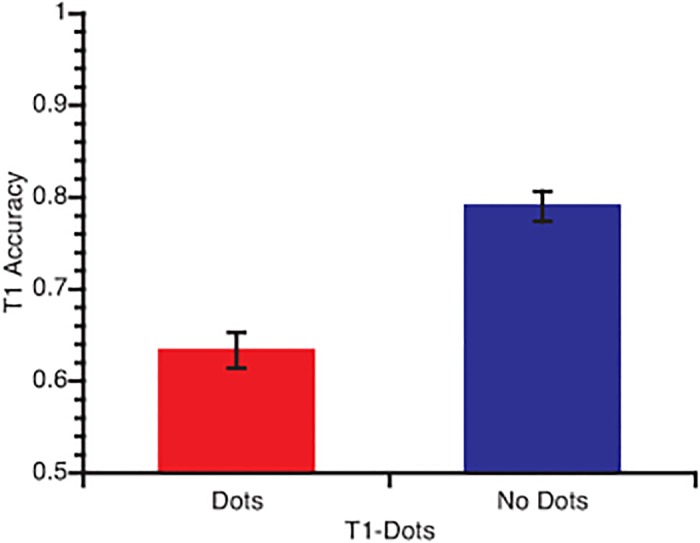
T1 accuracy as a function of T1-difficulty. Error bars in this and subsequent graphs represent standard error of the mean.

Mean T1 accuracy is plotted as a function of whether T1 was presented with or without dots in Fig 2. A repeated measures ANOVA revealed a main effect of T1-difficulty, such that accuracy was lower when T1 was presented with dots than without dots (F(1,50) = 140, p < .001, η^2^ = .737).

#### T2 Data

**Fig 3:**
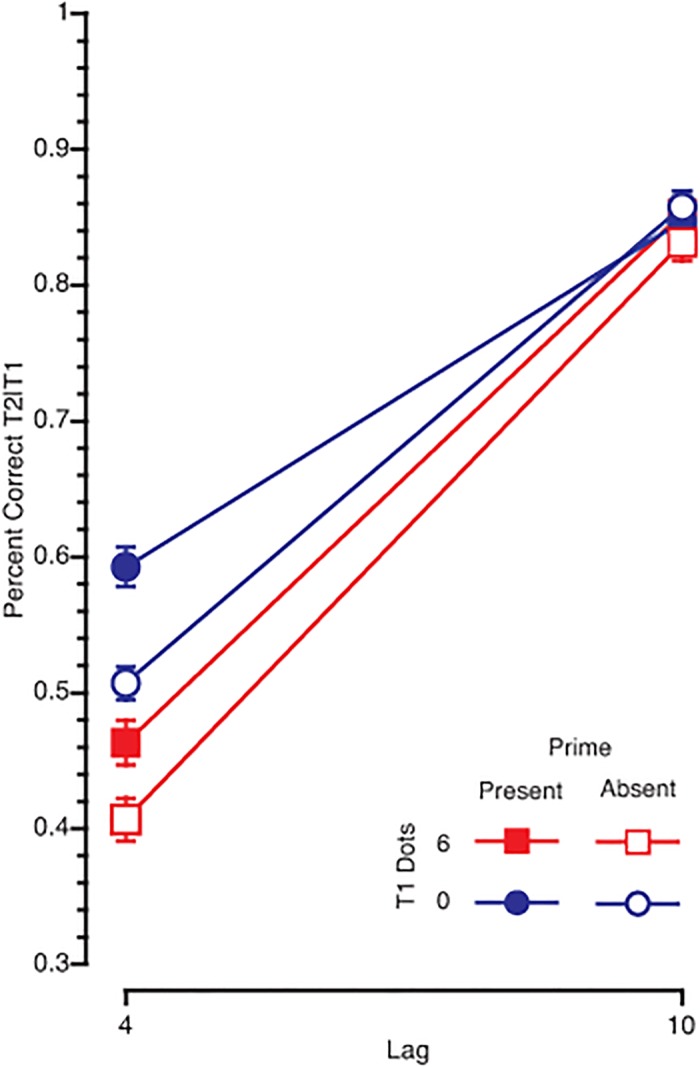
T2 accuracy. T2 accuracy, given correct report of T1, as a function of T1-difficulty condition and prime presence/absence.

Mean T2 accuracy on trials on which T1 was accurately reported (T2|T1) is shown in Fig 3 as a function of T1 difficulty and prime presence. The key finding for the present purpose was a three way interaction between prime, T1-difficulty, and lag (F(1,50) = 5.18, p = .027, η^2^ = .09). To determine the source of this significant three-way interaction, we examined the simple interactions at each level of T1-difficulty. When T1 was easy, there was a significant two-way interaction between prime and lag (F(1,50) = 26.63, p < .001, η^2^ = .35), such that the prime improved performance at short lags, but not long lags. However, this interaction was not significant when T1 was difficult (F(1,50) = 2.44, p = .125, η^2^ = .05). This suggests that the three-way interaction is driven by the influence that T1 difficulty has on the lag by prime interaction. Importantly, there was also an interaction between lag and difficulty, such that there was a larger AB when the dots were present compared to when the dots were absent (F(1,50) = 34.4, p < .001, η^2^ = .41).

There were 7 additional interactions or main effects in this design. The significant main effects included an overall increase in performance when the prime was present compared to when it was absent (F(1,50) = 22.3, p < .001, η^2^ = .31), a decrease in T2 accuracy in the difficult condition compared to the easy condition (F(1,50) = 69.9, p < .001, η^2^ = .583), and finally a decrease in performance at lag 4 compared to lag 10 (F(1,50) = 321, p <.001, η^2^ = .87). The effect of the prime was also more pronounced at lag 4 relative to lag 10 (prime x lag: F(1,50) = 17.1, p < .001, η^2^ = .255). However, there was not a significant two-way interaction between prime and difficulty (F(1,50) = .006, p = .94, η^2^ = .0001).

### Individual Differences in Distractor Processing and AB Magnitude

In order to replicate the previously reported individual differences in distractor processing and the AB magnitude [21], both the AB magnitude and the prime effect were calculated. However, unlike the previous work, the independence between these two measures (i.e., AB magnitude and the prime effect) was preserved by splitting the data (first half: blocks 1 through 5; last half: blocks 6 through 10). AB magnitude (lag 10 absent—lag 4 absent) and the prime effect (lag 4 present—lag 4 absent) were calculated for both the first half and the last half. Even though there was no correlation between the last half prime effect and the first half prime effect, there was a positive correlation between the first half AB and the last half AB (Table 1). There were no significant correlations between the prime effect and AB magnitude. Similar split-half correlations were conducted by splitting the data based on whether it was an odd or even trial. There were no significant correlations between the prime effect and AB magnitude (Table 1).

**Table 1:** Relationship between AB magnitude and the prime effect.

	odd AB	odd Prime	First 5 AB	First 5 Prime
**even AB**	0.347*	0.136		
**even Prime**	-0.168	0.169		
**Last 5 AB**			0.528**	0.191
**Last 5 Prime**			-0.176	0.222

Pearson correlation coefficient values examining the relationship between AB magnitude and the prime effect.

* p < .05

** p < .001

### Computational Simulation

**Fig 4:**
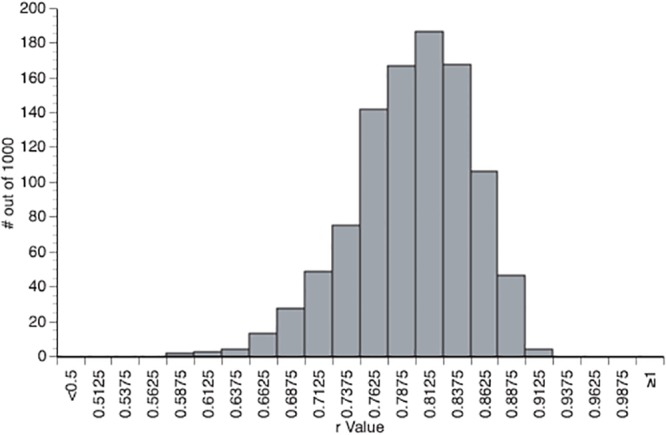
Distribution of Pearson correlation coefficients. Histogram of the Pearson’s correlation between the simulated AB and the simulated prime effect obtained from the 1000 randomly generated distributions with 48 data points.

A computational simulation was conducted in order to examine how calculating difference scores between a set of three variables influences the correlation between the difference scores. Specifically, forty-eight data points, equivalent to the number of subjects used in Dux and Marios [21], were randomly generated for each of the 3 conditions and one additional condition as a control. Each random number was drawn from a normal distribution with a mean and a standard deviation approximately equivalent to those reported by Dux and Marios [21]. The fourth control condition had a mean and standard deviation approximately equal to the lag 4 prime absent condition. Then the simulated lag 4 absent condition was subtracted from the simulated lag 4 present condition to simulate the prime effect (sPrime) and the simulated lag 4 absent condition was also subtracted from the simulated lag 10 absent condition to calculate the simulated lag 4 AB magnitude (sAB). Importantly, the simulated lag 4 absent condition is included in both calculations, as was done by Dux and Marios [21]. Finally, a simulated independent measure of AB magnitude was calculated by subtracting the simulated lag 10 absent condition from the control lag 4 absent condition. The relationship between sPrime and sAB was then examined by calculating the Pearson’s r and its associated p-value between the two values. Pearson’s r and p-values were also calculated to examine the relationship between simulated independent AB and sPrime. This was repeated 1000 times in order to get a population of r and p values. On average, the Pearson’s r between sPrime and sAB for the randomly generated data was .7964 (Fig 4) with a mean p-value of .000000056. In fact, all of the 1000 simulations were significantly correlated. However, the mean r-value for the relationship between sPrime and the simulated independent AB magnitude was-.0012 and the mean p-value was .5147. This clearly demonstrates that the shared variance induced by the calculations used for the prime effect and the AB magnitude effect in Dux and Marios [21] could completely explain the reported relationship between these variables.

## Discussion

The primary purpose of the present work was to test the flexibility of selective attention during the AB by investigating whether distractor suppression is modulated by task demands. The primary finding was that there was evidence for a decrease in the processing of information presented during the AB, as indexed by an overall decrease in the influence of the T2 prime under difficult T1 conditions. Previous studies have shown that a prime presented during the AB influences accuracy on the T2 task, clearly suggesting that the prime is extensively processed [17, 21, 33]. Thus, the present results support the notion that the noise dots presented simultaneously with T1 decreased the processing of the prime and that the extent to which information is processed during the AB is flexible. Furthermore, while other studies have shown that T1-difficulty can influence the magnitude of the AB [23–25], these studies have either included a task switch between T1 and T2 and/or a spatially distributed display. Therefore, it is possible that the influence of T1-difficulty on post-perceptual processing observed in those studies was due to the task switch and spatially distributed displays [13]. However, the current experiment eliminated the confounds of both a task switch between T1 and T2 and a spatially distributed display. Importantly, a robust influence of T1-difficulty on the severity of the AB was observed. These results suggest that the consequences of attending to T1, as indexed by T2 accuracy and the influence of the prime on T2 accuracy, are determined in part by the overall difficulty of the T1 task.

Previous work has highlighted the pitfalls of violating independence when examining correlations between neural activity and behavior in the neuroimaging literature [34]. These authors found that it was common practice to use the same data to examine correlations between neural activity and behavior as was used to define the regions of interest that were used in those correlations. Here we have highlighted another potential pitfall in examining individual differences by correlating two variables that have induced shared variance. Specifically, the results of the computational simulation and the attempt to replicate previous individual difference analyses clearly suggest that the relationship between the prime effect and AB magnitude reported in the previous studies [21, 30] could have been driven solely by the shared variance between these two variables induced by including lag 4 prime absent in both calculations. It is important to note that the task used by Dux and Marios [21] had a total of 200 trials. While the current experiment matched the number of trials per cell, the total number of trials was 400 because of the additional factor of T1-task difficulty. Thus, this increase in the number of trials might be an important factor that moderates the relationship between AB magnitude and distractor processing. Furthermore, it is important to emphasize that the present results do not completely rule out the possibility that a true correlation was present in the previously published work (i.e. [21, 30]). What the current results demonstrate is that an equally plausible explanation for the presence of the correlation observed by Dux and Marois [21] and by Slagter and Georgopoulou [30] is the manner in which the dependent measures were computed. Yet, it is important to note that other research clearly indicates that distractor processing influences the magnitude of the AB [12] and that the results reported here do not call this into question. What the present results suggest, however, is that distractor suppression measured using the priming manipulation used here and by previous investigators [21, 30] is not a reliable predictor of AB magnitude.

The finding that T1-difficulty decreases the processing of both distractors and targets is in line with other studies and suggests that the AB, at the very least, influences early perceptual processes. For instance, two papers have demonstrated that the difficulty of the T1 task influences target processing during the AB. Under easy conditions, it was found that the N400 to the T2 was robust inside and outside the AB [24], just like it was in the classic studies [20]. However, under difficult T1 conditions, the N400 evoked by T2 can be abolished [24]. In another study, Giesbrecht and colleagues [25] found that the severity of the AB for one’s own name was increased under difficult conditions, whereas previously it was found that one’s own name survives the AB [18]. Both of these experiments suggest that one factor that influences the extent to which information is processed during the AB is the difficulty of the T1 task.

While the present work fits well with these previous studies showing that T1 task demands can influence the extent to which information is processed during the AB, the precise mechanism(s) that mediates the effect of difficulty is unclear. For example, the effect of the noise dots used here can be interpreted within the context of accounts of simultaneous masking [35]. Simultaneous masks may not only increase the noise accompanying the T1 sensory signal, but also influence the processing of subsequent information either directly as a result of the noise lingering in the system or indirectly as a result of the engagement of inhibitory processes meant to dampen the noise, thereby decreasing the processing of subsequent information. This account may also explain the effects of the incongruent flankers used in previous studies [23–25, 27]. However, other mechanisms may also result in similar effects on the processing of the second target. For example, competition between stimuli at the level of single unit receptive fields has been proposed as a mechanism to explain the reduction in the extent to which task irrelevant information is processed during visual search [36, 37] and this concept may provide an account of the effect of T1 task difficulty on the processing of information during the AB.

Other studies that have not included a manipulation of T1 task difficulty have also suggested that the consequences of selecting the T1 are more severe than previously thought. For instance, Jolicoeur and colleagues [38] found that the N2PC, an ERP component associated with directing attention to a target amongst distractors [39], was suppressed during the AB. Given that the N2PC is thought to represent selection of information at a perceptual level [39], this finding suggests that the AB involves a failure of selection at a relatively early level. Furthermore, Vachon and Jolicoeur [28] found that if there is a task switch between T1 and T2, then the magnitude of the semantic mismatch negativity (N400) is also reduced, which suggests that processing does not always proceed to the post-perceptual level during the AB.

Based on the results of the current experiment and other experiments in the literature, it appears that the consequences of selecting and/or consolidating T1 during an RSVP task are more severe than previously demonstrated. Specifically, the assumption that all information presented during the AB is processed to the level of semantic and or categorical representation (e.g. post-perceptually), which is held by almost all theories of the AB, is unwarranted. It is also clear that at least three different manipulations determine the level of processing during the AB: 1) T1 task difficulty [23–25], 2) task switches between the T1 and T2 [28], and 3) spatial location of T2 [22]. While each of these manipulations influence the extent to which subsequent information is processed, the mechanism by which these manipulations do so likely varies. Nonetheless, it is important for theories of the AB to take into account early perceptual processes as a potential factor in causing and modulating the AB. Importantly, we are not suggesting that the AB is caused solely by failures at an early perceptual level, but rather that perceptual processes can contribute and interact to create and modulate the AB. Independent of the actual cause of the AB, our results and those in the literature clearly demonstrate that the extent to which information is processed during the AB is contingent on the structure and difficulty of the task. Therefore, the AB does not represent a phenomenon that is fundamentally unique, but represents the more general perceptual and cognitive dynamics of attention.

## Supporting Information

S1 Appendix Mathematical Discussion of Inflated Shared Variance. (PDF)

S1 File Data. Archived (.zip) file of data (in excel spreadsheets) for current experiment.
(ZIP)
