# Supplementary material for: Distractor Suppression When Attention Fails: Behavioral Evidence for a Flexible Selective Attention Mechanism
Source: PLoS One. 2015 Apr 27;10(4):e0126203. doi: 10.1371/journal.pone.0126203 (PMC4411164; doi:10.1371/journal.pone.0126203)
Supplement: S1 Appendix — (PDF) [file pone.0126203.s001.pdf]

S1 Appendix.

Mathematical Discussion of Inflated Shared Variance

Suppose A, B, and C are the distributions for three independent random variables representing the three conditions included in the prime effect and AB magnitude calculated by Dux and Marios (2008). In the current example, B would be equivalent to the lag 4 prime absent condition, which was included in both calculations. The prime effect and AB magnitude calculations are represented by X and Y, such that X = A – B and Y = C – B. Then, by definition the Pearson's correlation is (for definitions and properties of correlation, variance, and covariance see Rice, 1995):

$$\mathrm{Cor}(X,Y) = \frac{\mathrm{Cov}(X,Y)}{\sqrt{(\mathrm{Var}(X)\mathrm{Var}(Y))}} \quad (1)$$

Substituting A – B for X and C – B for Y:

$$= \frac{\mathrm{Cov}(A\text{-}B,C\text{-}B)}{\sqrt{(\mathrm{Var}(A\text{-}B)\mathrm{Var}(C\text{-}B))}} \quad (2)$$

Given the bilinearity of covariance:

$$= \frac{\mathrm{Cov}(A,C) - \mathrm{Cov}(B,C) - \mathrm{Cov}(A,B) + \mathrm{Cov}(B,B)}{\sqrt{[\mathrm{Var}(A)+\mathrm{Var}(B)][\mathrm{Var}(C)+\mathrm{Var}(B)]}} \quad (3)$$

Since the covariance between two independent random variables, by definition, is 0, and the covariance of a random variable with itself is the variance of that random variable:

$$= \frac{\text{Var(B)}}{\sqrt{[\text{Var(A)}+\text{Var(B)}][\text{Var(C)}+\text{Var(B)}]}} \tag{4}$$

Thus, the shared variance in the correlation between X and Y is largely determined by the variance of B. In the case where the variance of B is equal to the variance of A and C, then the expected value of the correlation would be .5. To the extent that the variance of B is greater than the variance of A and C, then the expected value of the correlation is at least .5 and approaches 1. On the other hand, if the variance of B is substantially less than the variance of A and C, then the expected value of the correlation is less than .5 and approaches 0. Based on the information available in Dux and Marois (2008), the lag 4 prime absent had a variance of about .0299 and the other two conditions had variances of about .00488. If one were to assume no correlation between the variables, then based on these values one would expect the r-value for the correlation between the difference scores to be .8621.

Works Cited

Rice, J. A. (1995). *Mathematical Statistics and Data Analysis* (2nd Edition ed.). Belmont, California: Duxbury Press.
